# Sustainable Data-Driven Secured Optimization Using Dynamic Programming for Green Internet of Things

**DOI:** 10.3390/s22207876

**Published:** 2022-10-17

**Authors:** Tanzila Saba, Amjad Rehman, Khalid Haseeb, Saeed Ali Bahaj, Robertas Damaševičius

**Affiliations:** 1 Artificial Intelligence and Data Analytics (AIDA) Lab, CCIS Prince Sultan University, Riyadh 11586, Saudi Arabia; 2 Department of Computer Science, Islamia College Peshawar, Peshawar 25120, Pakistan; 3 MIS Department, College of Business Administration, Prince Sattam bin Abdulaziz University, Alkharj 11942, Saudi Arabia; 4 Faculty of Applied Mathematics, Silesian University of Technology, 44-100 Gliwice, Poland

**Keywords:** sustainable computing, optimization, Internet of Things, blockchain, edge computing, green process, technological development

## Abstract

The development of smart applications has benefited greatly from the expansion of wireless technologies. A range of tasks are performed, and end devices are made capable of communicating with one another with the support of artificial intelligence technology. The Internet of Things (IoT) increases the efficiency of communication networks due to its low costs and simple management. However, it has been demonstrated that many systems still need an intelligent strategy for green computing. Establishing reliable connectivity in Green-IoT (G-IoT) networks is another key research challenge. With the integration of edge computing, this study provides a Sustainable Data-driven Secured optimization model (SDS-GIoT) that uses dynamic programming to provide enhanced learning capabilities. First, the proposed approach examines multi-variable functions and delivers graph-based link predictions to locate the optimal nodes for edge networks. Moreover, it identifies a sub-path in multistage to continue data transfer if a route is unavailable due to certain communication circumstances. Second, while applying security, edge computing provides offloading services that lower the amount of processing power needed for low-constraint nodes. Finally, the SDS-GIoT model is verified with various experiments, and the performance results demonstrate its significance for a sustainable environment against existing solutions.

## 1. Introduction

IoT is a network of “things” that share and collect data from the environment. These “things” could be sensors, portable electronics, wearable technology, or any networked item that can carry out certain tasks [1,2,3]. Similarly, a Wireless Sensor Network (WSN), as a component of IoT, transfers the obtained data after detecting any incident. However, there is limited security in the IoT environment due to its scale and heterogeneity, making it vulnerable to various assaults, including WSN-inherited attacks [4,5,6]. Sustainable computing has been extensively employed in social networks and IoT in the previous decade [7,8,9]. IoT has social, economic, and commercial consequences on human life. However, the open channel, i.e., the internet and IoT nodes used for data transmission channels, is vulnerable to a wide range of intrusions and routing processes. Several initiatives are underway in this area to cope with the developing security challenges in IoT systems and make them self-sufficient in harvesting energy for smooth operation [10,11]. Cloud computing is essential in contemporary culture and allows various sustainable applications, ranging from infrastructure to social media [12,13,14]. Guarantees of Quality of Service (QoS) must be met by such a system, which must be able to handle variable loads and use patterns that reflect the interaction and reliance of societies on automated computer systems [15,16,17]. These systems are enabled by a group of conceptual technologies that have been synthesized to fulfill the requirement of growing computer applications. However, with the increased proliferation of IoT devices, concerns such as security, privacy, efficiency, and environmentally friendly computing infrastructure are growing daily [18,19]. Therefore, to develop a sustainable computing environment for future smart cities, it is necessary to consider their whole life cycle, from design to production for recycling and disposal, as well as their broader influence on people and places. Additionally, the system should consider the vulnerable attacks on IoT networks with strong privacy and authentication techniques [20,21].

The proposed SDS-GIoT model offers the following contributions.

- It provides a data-driven approach for sustainable smart cities using multistage graph-based structures and improves the system’s response time.
- Intelligent decisions are made based on dynamic programming, which allows for effective computing with minimum complexity on the IoT networks.
- Edge computing and deterministic technique are combined to create and maintain system security. Using an offloading method lessens the burden of security measures on the devices.
- The proposed SDS-GIoT model is verified in terms of green computing metrics against existing work.

This research paper is structured in sections. Section 2 discusses the literature work. The SDS-GIoT model, with its developed components and states, is explained in Section 3. The network model and performance analysis are described in Section 4. Finally, Section 5 concludes with a summary.

## 2. Literature Review

The development of information and communication technologies over the past few decades has sparked a trend toward smartening everyday things to improve human comfort. The paradigm of smart cities is a reaction to the objective of building future cities for quality assurance and sustainable development [22,23,24]. Smart devices and dynamic wireless systems offer various benefits for developing heterogeneous networks. Using these kinds of technologies, smart sensing, network automation, and resource management is made feasible around us. However, due to the energy and other constraints on IoT devices, researchers are still searching for recommended green solutions for IoT networks. Another important research goal is to ensure the safety of network data from potential threats [25,26]. In [27], the authors presented a power-efficient tree-based routing algorithm that decreases end-to-end latency in energy-efficient green-IoT networks with a mobile sink. The proposed protocol offers two new distinct ways of controlling network routing. The first mechanism uses a more dependable and energy-efficient version of the geographic routing algorithm. The second mechanism uses a tree-based structure, which can be built with the fewest possible control packets and updated efficiently. According to simulation findings, the proposed routing protocol is superior to the existing solutions in terms of energy consumption, network longevity, delay, and throughput.

The authors in [28] developed a Mobility-Aware Dynamic Clustering-based Routing (MADCR) protocol for the Internet of Vehicles (IoV) to optimize the lifetime of networks and decrease the end-to-end communication latency. The MADCR protocol includes methods for cluster formation and Cluster Head (CH) selection. The formation of a cluster is based on Euclidean distance. The CH is then selected using the Mayfly Optimization Algorithm (MOA). The CH then receives vehicle data and transmits this information to the Road Side Unit (RSU). In addition, the proposed MADCR protocol reduced the end-to-end delay and improved the packet delivery ratio as compared to other studies. Authors in [29] proposed a Secure Routing Protocol by using Multi-objective Ant-colony-optimization (SRPMA) for WSN. They upgraded the ant colony method to be a multi-objective routing algorithm using the residual energy of nodes. First, a routing path is formed by exploring multi-pheromone and multi-heuristic information. The node trust assessment model enhances the D-S evidence concept with conflict preparation. Second, multi-objective routing results were generated by employing crowd distance criteria.

A novel routing protocol termed Secured QoS aware Energy Efficient Routing Protocol is presented to improve WSN security and energy efficiency [30]. Trust modeling employed a key-based authentication approach to provide trust ratings in this suggested study. This study calculated direct, indirect, and total trust ratings to improve communication security. They also proposed a cluster-based safe routing method where the cluster head is chosen based on QoS measurements and trust ratings. The ultimate secure routing route is based on path trust, energy, and hop count. The Software-Defined Network (SDN) approach has recently been combined with IoT to address prospective scalability and flexibility needs to create SDN-IoT. As SDN-IoT grows, efficient routing methods with low latency and robust security are required. However, the default SDN routing protocols are subject to dynamic flow control rule changes when the network is under attack. The Internet of Things Cloud (IoTC) may enhance the sensing capabilities of IoT devices, while fog computing can circumvent IoT devices’ processing and storage constraints. Accordingly, the authors [31] proposed a fog-assisted IoTC data collecting and delivery architecture to minimize IoTC-based data transfer costs and increase success rates. Their framework utilized named data networking to provide IoTC-based data, allowing numerous IoT devices to obtain data from the closest provider through a single data delivery mechanism. In addition, the framework provided mobility support for IoT devices to ensure adequate data reception. They employed a neuro-fuzzy rule-based clustering technique for cluster-based routing. In their method, the cluster creation in WSNs used energy modeling to effectively route packets using machine learning with a convolutional neural network and fuzzy rules for weight modification, extending the network lifespan. As a result, their routing algorithm exhibited better efficiency in terms of usage, packet delivery ratio, latency, and network lifespan.

The technology of IoT networks with SDN architecture is being explored for the formation of smart cities. It senses the data from the targeted area and sends it to remote sites for processing and analysis. Many solutions have been recommended by researchers based on the aforementioned discussed work for coping with resource constraints with computing capabilities; however, the management of network data over the presence of huge traffic flow is one of the main research problems. Moreover, providing prompt responses on remote sites with affordable delay is also considered a significant research problem. It was also noticed that optimizing the routing process coped with the support of intelligent strategies; however, it exposed additional costs to the IoT devices as the network grew. This research proposed a reliable model for IoT technologies with the support of dynamic programming, leading to optimized efficiency. The states of the SDS-GIoT model are evaluated each time before announcing the optimal decision for data sending. Furthermore, edge computing integrates with blockchain technology to ensure data privacy and authentication. Edge computing provides an offloading method for decreasing the network burden in terms of applying security on the constraint devices.

## 3. Sustainable Data-Driven Secured Decision Protocol with Dynamic Programming 

In this Section, we present the system model and detail of the SDS-GIoT model. The SDS-GIoT model comprises sensors, edge nodes, IoT devices, and wireless systems. IoT devices and sensors are utilized for collecting and transmitting environmental data. IoT devices and sensors explore the proposed routing algorithm for sustainable communication with minimum data damage. Additionally, the multi-variable objective function helps link predictions and facilitates dynamic programming for optimal decisions. The data is routed from the consistent and reliable nodes toward edge devices based on the decisions. The decisions are dynamic because they change each time the source node needs to send Route Request (RREQ). Later, the security layer is used to identify the potential threats in the IoT system. The main sub-sections of the SDS-GIoT model are discussed as follows.

### 3.1. System Model 

The proposed network model consists of sensors interacting with each other using wireless communication standards. The sensors denote by $X_{i}$, where i = 0, 1, 2, …, *n*. The nodes are arranged in the form of directed and weighted multistage Graph $G\left(N,\;E\right)$, so adjacent nodes are directly connected using edge E. Each stage is comprised of various sensor nodes. Additionally, edges have a weight known as cost and are updated on certain conditions. All the devices have some kind of restrictions regarding resources, especially energy, transmission power, and memory. Edge computing performs the offloading services in terms of security analysis and ultimately decreases the additional overhead of the nodes. The edge devices are more powerful than ordinary nodes. Following are some network assumptions for the SDS-GIoT model.

- The sensor nodes can only communicate with edge devices and are not mobile.
- There are no resource restrictions on the network edges or sink nodes.
- At the edge of a sensor’s vicinity, edge devices are randomly positioned.
- No more nodes or devices can be included after deployment.
- Malicious nodes can generate false information and compromise the sending data and wireless channels.

### 3.2. States of the SDS-GIoT Model 

**Figure 1:**
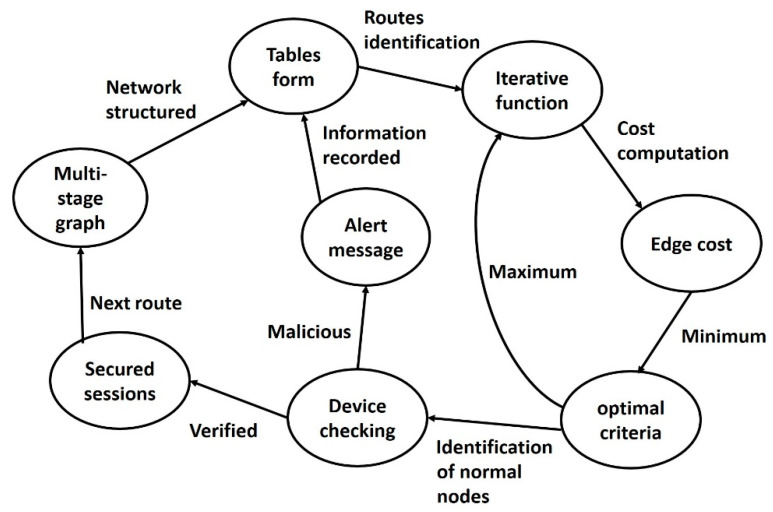
States of the SDS-GIoT model.

This Section provides a brief introduction to the states in the SDS-GIoT model. Figure 1 depicts the various states and their association with each other.

- Multistage graph: This stage organizes the nodes in the form of multiple stages, and stages are interconnected with edges.
- Tables’ initialization: In this state, nodes’ information and network conditions are recorded along with the identities of devices.
- Iterative function: The repeated function is performed to determine the optimal routing strategy in this state.
- Edge cost: Nodes compute the cost, and accordingly, the minimum value offers the optimal decisions. In case the outcome is not optimal, then the iterative function is executed again.
- Authentic nodes: This state determines the validity of devices in terms of authentication. If nodes are declared authentic, then communication is allowed by the system; otherwise, alert messages are recorded in the local tables.
- Secured sessions: In this state, the system attains data privacy with integrity using session keys and security methods.

### 3.3. Model Discussion 

In this Section, we present a novel solution for routing the IoT data with the support of dynamic programming. The sensors sense the surrounding data and execute the next-hop selection process in a distributed way, unlike most of the proposed solutions that do not track the congestion and traffic-related information in the decision system. Our proposed data-driven model keeps such information while reaching an optimal decision. Accordingly, the SDS-GIoT model offers a sustainable approach to attaining a timely wireless system with load balancing. In the SDS-GIoT model, the nodes are arranged in the multistage graph such that each node has some neighbors until it reaches the destination state. We consider states are denoted by $S_{i}$ that comprised on $N_{i}$ and cost $C_{i}$. The nodes in the same stages cannot communicate with each other. The objective function can be computed by exploring dynamic programming as given in Equation (1).

(1) $$f\left(S_{i},\;N_{i}\right)\to \;\;(S_{i+1},K)\;:\;\min\;C\left(N_{i},K_{i}\;\right))$$

subject to:

$$K\;\varepsilon \;S_{i+1}$$

In Equation (1), $S_{i}$ is the current stage, $N_{i}$ denotes visited vertex, $S_{i+1}$ is the next stage, and K denotes the visited vertex in the next stage. $C\left(N_{i},K_{i}\right)$ represents the cost from the vertex $N_{i}$ to vertex $K_{i}$. It is an iterative process and is executed until achieving optimal end-to-end routing performance. The SDS-GIoT model performs the main role by cost value to select the optimal path. Unlike most of the existing approaches that choose the routes without adopting the realistic factors for the formulation of the route, the SDS-GIoT model explores traffic prediction for the computation of cost value. With the support of traffic prediction TP, the SDS-GIoT model efficiently identifies the most optimal link for forwarding IoT data. Moreover, along with traffic prediction, it also integrates the priority of link status LS for nodes i, j, as given in Equation (2).

(2) $$C\left(i,\;j\right)\;=\;TP+\;LS$$

To evaluate priority Pr for LS, the SDS-GIoT model utilizes the waiting time and packet reception information, as defined in Equation (3).

(3) $$Pr\left(LS\right)\;=no.\;of\;packets/waiting\;time$$

A connection is not taken into account by the decision-making algorithm if the Pr of LS is less than a certain threshold. In Equation (3), if waiting time increases, then, accordingly, the priority for a specific link decreases. On the other hand, to determine the Pr for TP on the communication link, the SDS-GIoT model utilizes bandwidth B and queued packets P as Equation (4).

(4) $$Pr\left(TP\right)\;=B/P$$

According to Equation (4), when the queued packets increase, the priority value of TP decreases. Also, if TP is below a specified threshold, the decision-making mechanism excludes that particular communication channel. In the SDS-GIoT model, each node establishes and maintains its routing table and updates it when certain conditions occur. All the nodes are associated with their edge devices and can be monitored intelligently. Whenever residual energy e of any forwarded node fn is less than the predefined threshold, then the edge device initiates the request to the source node for route re-formulation, as defined in Equation (5).

(5) $$e\left(fn\right)\;<\;threshold;RREQ=true$$

**Figure 2:**
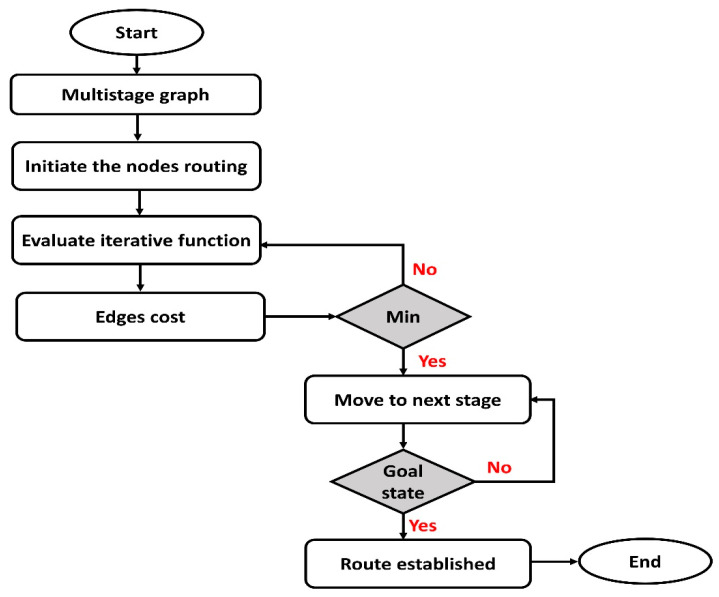
Flowchart of proposed routing algorithm using dynamic programming.

Figure 2 depicts the flowchart of the SDS-GIoT model for sustainable routing using dynamic programming. The main phases are multistage graphs, initiate iterative routing, cost function, and route updating. In the beginning, nodes are arranged in multistage graphs and divide the network structure into stages. Then, the source node needs to determine the optimal node in the next stage dynamically, and this process is iterative until data are delivered to their destination. Moreover, the cost function is a key parameter in achieving a sustainable and efficient routing scheme. The cost function is based on the traffic load and link status; accordingly, the routing phase can be re-formulated dynamically, and information in the routing tables is updated. 

Afterward, the SDS-GIoT model develops a security algorithm for devices with limited constraints. In this phase, edge devices act as a central hub for their associate sensors, and they help authenticate the peer devices before data transmission. Let us consider that i and j are sensor nodes that need to collaborate for data routing. The edge device is denoted by ED, E is any symmetric encryption algorithm. Firstly, i intends to communicate with ED and j. Protocol messages are initiated, and security functions are applied accordingly to achieve this. Then, i encrypts message D using $k_{i,ED\;}$ along with the identity of the intended recipient $id_{j}$, and sends this to ED with its identifier $id_{i},$ as defined in Equation (6).

(6) $$i\to ED:k_{i,ED\;}\left(id_{j,\;\;\;}D\right)+id_{i}$$

Upon decrypting the message, ED determines it is intended for j, looks up the $k_{j,ED}$ of the indicated recipient, and re-encrypts D for j. Later, ED returns the translated message for i to send towards j, as defined in Equations (7) and (8).

(7) $$ED\to i:k_{j,ED\;}\left(id_{i,\;\;\;}D\right)\;+\;t$$

(8) $$i\to j:k_{j,ED\;}\left(id_{i,\;\;\;}D\right)\;+\;t^{\prime }$$

where t and $t^{\prime }$ denote time stamps to avoid reply attacks. Once nodes are identified as authentic, they initiate the data transmission process with the support of blockchain. In this phase, nodes generate their new session keys and share them with associated devices for communication. ${k_{i\;}}^{\prime }$ and ${k_{j\;}}^{\prime }$ are generated session keys for nodes i and j. Afterward, both devices send the keys with encryption using associated private keys assigned by the edge device. The process for exchanging keys using the encryption function is defined in Equations (9) and (10).

(9) $$i\to j:k_{j,ED\;}\left({k_{i\;}}^{\prime }\right)$$

(10) $$j\to i:k_{i,ED\;}\left({k_{j\;}}^{\prime }\right)$$

Later, using received keys, the SDS-GIoT model performs data encryption on data blocks $B_{i}$ as defined in Equation (11).

(11) $$i\to j(xor(B_{i},\;{k_{i\;}}^{\prime });j\to i(xor(B_{i},\;{k_{j\;}}^{\prime })$$

**Figure 3:**
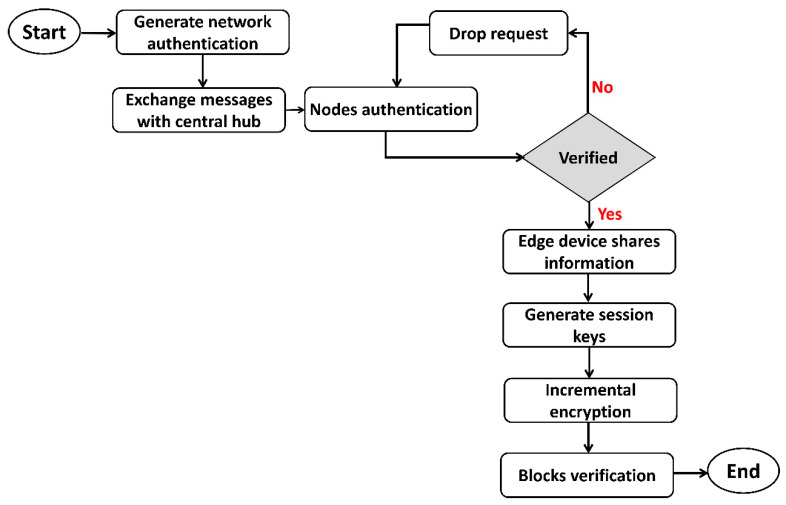
Flowchart of the proposed security algorithm using authentic methods.

This process continued in the form of various increments for maintaining the integrity and privacy of data blocks. Figure 3 illustrates the flowchart of the proposed security algorithm. Initially, network authentication is performed with the intelligence of the central hub. Edge devices perform offloading security functions with their processing capabilities, and only lightweight operations are allowed in constrained devices. When nodes are declared authentic, they generate session keys to initiate the data routing with their associated devices. Also, session keys are encrypted with the private key of nodes that are distributed by edge devices. Later, data blocks are encrypted incrementally by exploring blockchain technology. Algorithm 1 shows the pseudocode in the development of the SDS-GIoT model.
**Algorithm 1**: Data-driven secured optimization model using dynamic programmingStep 1: *Procedure* Sec_data_driven Step 1: Network-setup Step 2: Multistage graphs with nodes and edges Step 3: Compute the traffic by exploring TL = B/P Step 4: Compute $Pr\left(LS\right)\;$= no. of packets/waiting time Step 5: Cost using objective function $C\left(i,\;j\right)\;$= TL + LS Step 6: Threshold evaluation for sending route request $e\left(fn\right)$ < threshold;RREQ=true Step 7: *If* the neighbor state is not equal to the destination then Repeat Steps 3 to 6 *End if* Step 8: Performs network authentication Step 9: *If* authenticity is verified then data transmission *Else* Drop the request packet *End if* Step 10: Generate random keys and perform a security function Step 11: *If* all data packets are delivered to the destination then Send ACK to the source device *Else* Perform incremental encryption *End if* Step 12: *End procedure*

## 4. Simulations

This Section describes the network scenarios and the performance results of the SDS-GIoT model with MADCR and SRPMA. We perform various tests in two scenarios. One is varying IoT devices and the second is varying data generation rates. IoT devices range in number from 25 to 125, and data generation rates range from 1000–5000 bits/s. The varying number of nodes is considered in experiments to identify the effectiveness of the SDS-GIoT model under different sizes. The data generation rate indicates the amount of data that is produced by the nodes with time. The size of the control packet is set to 256 bits. We consider 10 edge devices and 2 sink nodes. The initial energy of nodes varies from 3 j to 6 j. The distance between nodes is measured using Euclidean distance. All the devices have limited constraints except edge devices and sink nodes. The simulations are carried out using NS-2.35 on the Ubuntu platform. The network dimension is set to 1000 m × 1000 m. A total of 30 simulations were run to take an average of the performance metrics. The transmission power of the IoT nodes is set to 10 m. Furthermore, varying malicious nodes are deployed in the simulation environment. The data were obtained from trace files after various simulations, and stored in log files for further analysis and determining the efficacy of the SDS-GIoT model. Table 1 mentions the parameters list with their default values for simulation. 

### Results and Discussion

**Figure 4:**
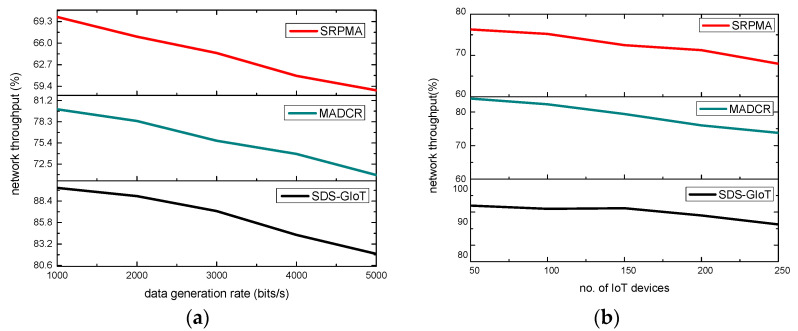
Scenarios for varying IoT devices and data generation rates in terms of network throughput. (**a**) network throughput and IoT devices. (**b**) network throughput and data generation rates.

This Section provides the simulated graphs and their discussion of the SDS-GIoT model and other related studies. The performance is evaluated for network throughput, latency, packet drop ratio, and the maximum number of rounds in a green IoT system. The proposed SDS-GIoT model remarkably improved green computing using the dynamic structure and the least overhead. It consumes less energy in sensing, aggregation, and transmission phases with the support of intelligent decision systems. The usage of multistage graphs explicitly shows a better outcome for the proposed sustainable solution in the light of dynamic programming. Moreover, the authentication and security operation also protect the green system by exploring lightweight cryptographic operations. The SDS-GIoT model maintains the efficiency of IoT resources in terms of energy constraints and increases its performance as compared to existing work. Unlike the majority of the existing work, which does not consider the intelligent approaches for the identification of suitable nodes and links in the data forwarding processes, to determine the most reliable circumstances and make the IoT system more sustainable, the proposed SDS-GIoT model makes use of dynamic programming. In Figure 4a,b, the performance of the SDS-GIoT model is compared with other work in network throughput. It can be defined as successfully transmitting sensor data packets to sink nodes. Based on the experiment’s results, it is clear that the SDS-GIoT model greatly increased network throughput by an average of 22% for varying IoT devices and 27% for varying data generation rates. This is because the dynamic programming is by the SDS-GIoT model and identifies the most optimal routes for forwarding the IoT systems. Its link prediction significantly increases the chosen routes’ strength and improves the system’s performance. Moreover, the boundary of the edges collaborates with the sink node and reduces the security overhead for limited constraint devices. Thus, it balances the energy depletion for sensors and explicitly provides the most stable communication link for the delivery of data.

**Figure 5:**
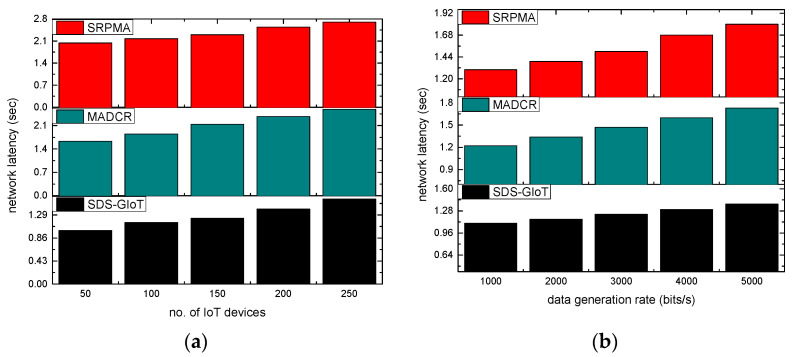
Scenarios for varying IoT devices and data generation rates in terms of network latency. (**a**) network latency and IoT devices. (**b**) network latency and data generation rates.

The performance of the SDS-GIoT model is evaluated in comparison with existing approaches. Figure 5a,b illustrates the performance of network latency, and it has been noticed that as nodes and data generation rates grow, the latency ratio is also increased. According to the experimental findings, the SDS-GIoT model has improved the latency ratio by an average of 21% in terms of varying IoT devices and 24% in terms of varying data generation rates. It results from the effective cost function evaluation and extracts the robust routes from the multistage graphs. Additionally, dynamic programming chooses nearby states that place the least burden on communication and trains the system with timely delivery. By utilizing the blockchain and authentic techniques, the SDS-GIoT model eliminates the most malicious attacks on the links and accordingly provides efficient forwarding of IoT messages.

**Figure 6:**
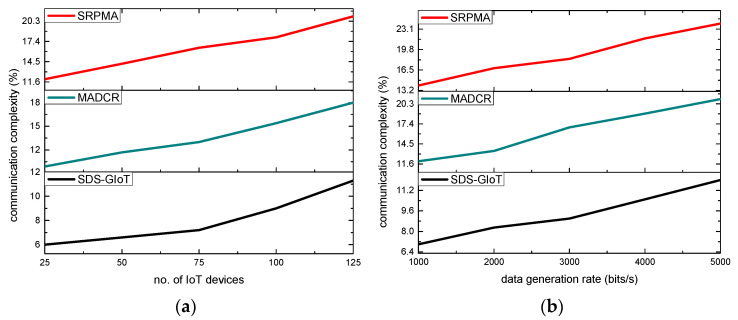
Scenarios for varying IoT devices and data generation rates in terms of communication complexity. (**a**) communication complexity and IoT devices. (**b**) communication complexity and data generation rates.

The performance of the SDS-GIoT model in comparison to the existing approaches is shown in Figure 6a,b. According to the research, the ratio of communication complexity also increases when the number of nodes and data generation rates have increased. However, the SDS-GIoT model improved the complexity rate by an average of 23% and 27% in terms of varying IoT devices and data generation rates as compared to other existing work. It is due to efficiently managing network resources and training the system using dynamic programming to avoid unreliable nodes. Additionally, the nodes are not frequently generated by the routing requests until and unless the energy level crosses the predefined threshold. Furthermore, unlike most of the existing work, the communication links are monitored regularly, and whenever any malicious action is performed, appropriate alert messages are recorded in the forwarding tables. Accordingly, the SDS-GIoT model reduces the affected data packets over the wireless medium. As a result, the SDS-GIoT model efficiently tackles the misbehaving threats from the nodes and decreases the communication complexity for data transmission and resources management.

**Figure 7:**
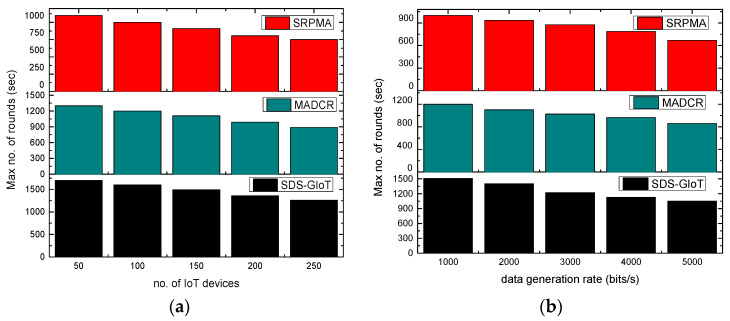
Scenarios for varying IoT devices and data generation rates in terms of no. of rounds. (**a**) Max no. of rounds and IoT devices. (**b**) Max no. of rounds and data generation rates.

Figure 7a,b illustrates the experimental results of the SDS-GIoT model as compared to existing work in terms of the number of rounds. It was noticed that the number of rounds decreases with the message of time. It is due to the excessive energy consumption of the nodes and frequently generated data packets. However, it is revealed that the SDS-GIoT model improved the network’s lifetime by an average of 42% and 32% in terms of varying IoT devices and data generation rates. It is due to the following aspects. Firstly, intelligent computing for developing a sustainable system increases the strength of the chosen routes and efficiently balances the load distribution. Secondly, dynamic programming always tries to attempt the most reliable forwarders to route the system data toward the sink node using edge efficacy. Additionally, the dynamic decision effectively prevents the system from entering the incorrect states and controls the flooding of fake control packets. Such a process leads to reduce complexity and improves the system’s lifetime.

**Figure 8:**
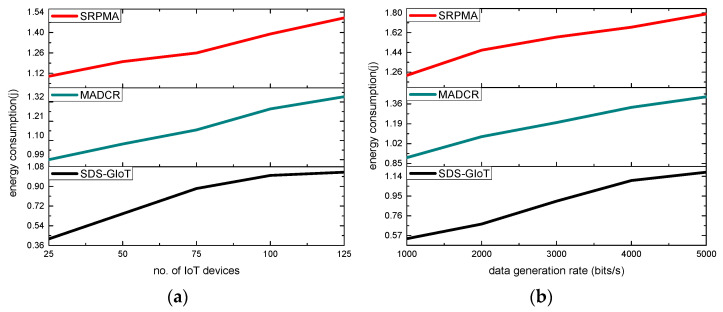
Scenarios for varying IoT devices and data generation rates in terms of energy consumption. (**a**) energy consumption and IoT devices. (**b**) energy consumption and data generation rates.

A comparison of the SDS-GIoT model with alternative studies is shown in Figure 8a,b. According to the revealed results, it was observed that the SDS-GIoT model significantly increases the efficient utilization of energy resources of the nodes by an average of 31% and 30% in terms of varying IoT devices and data generation rates. It has been found that as the IoT network grows, the network medium is also busy forwarding the huge nodes’ data, which greatly decreases the performance of the network in terms of energy consumption. On the other hand, the SDS-GIoT model provides an intelligent solution using dynamic programming and explores the routes in the graphs for the formulation of the routing phase. Additionally, edge computing balances the load distribution for its closer nodes and transmits the data on time. The blockchain-based technology in the SDS-GIoT model also avoided the probability of route breaches and minimized the additional energy consumption in frequent route requests.

## 5. Conclusions

Recently, the development of a sustainable IoT system solution using a combination of storage and communication devices has experienced rapid growth. Although IoT nodes are self-configuring and inexpensive, they have energy, transmission, and memory limitations. Most of the solutions have been vulnerable to numerous network threats and compromised information as a result of faulty communication connectivity. This paper presents a communication protocol for an IoT-based system using dynamic programming in multistage graphs and extracts the optimal information for the transmission of network data. Additionally, it decreases the response time and efficiently manages the nodes’ energy resources. Additionally, the proposed security mechanism is controlled by edge devices without additional overhead to the IoT nodes. The secret keys and data blocks are securely forwarded toward the sink using authentic blockchain technologies. The simulation results have proven the efficacy of the proposed protocol with related studies in terms of performance metrics. However, it was observed that moving the IoT nodes from their initial deployment points causes frequent data interruption and connectivity issues. As a result, the loss rate of packet reception increases. In the future, we aim to improve the proposed protocol in terms of the mobility model and would like to integrate deep learning intelligent techniques for coping with distributed network attacks.

## Figures and Tables

**Table 1:** List of simulation parameters.

Parameters	Values
Simulation area	1000 m × 1000 m
Devices distribution	Random
IoT devices	25–125
Data generation rates	1000–5000 bits/s
Transmission power	10 m
Initial energy	3–6 j
Simulations	30
Round interval	20 s
Data flow	CBR
Sink node	2
Edge nodes	10
Size of control packet	256 bits

## Data Availability

All data is available in the manuscript.
